# What causes concordance of hypertension between spouses in India? Identifying a critical knowledge gap from a nationally representative cross-sectional sample of 63,020 couples aged 15 + years

**DOI:** 10.1186/s12889-023-16379-z

**Published:** 2023-07-27

**Authors:** Gayatri Nayak, Shishirendu Ghosal, Jyoti Ghosal, Ambarish Dutta

**Affiliations:** 1 Tata Steel Foundation, Angul, Odisha India; 2 Indian Council of Medical Research—Regional Medical Research Centre, Bhubaneswar, Odisha India; 3 KIIT School Public Health, KIIT Deemed to Be University, Bhubaneswar, Odisha India; 4 Indian Institute of Public Health, Public Health Foundation of India, Bhubaneswar, Odisha 751013 India

**Keywords:** Concordance, Environment, Hypertension, Spouses, India

## Abstract

**Background:**

Hypertension, a critical risk factor for cardiovascular diseases, is found to cluster between spouses due to within-couple aggregation of antecedent environmental risk factors, either through assortative mating or cohabitation. However, majority of the evidence of spousal concordance of hypertension is from Caucasoid couples from western societies, whereas marriage, partner selection, and post-marital roles of husband and wives are very different in Indian society. Therefore, we aimed to comprehensively examine the phenomenon of spousal concordance of hypertension in Indian couples.

**Method:**

Couples from Longitudinal Ageing Study in India Wave 1 (*n* = 10,994) and National Family Health Survey Round 5 (*n* = 52,026) represented 15 years + Indian spouses. Hypertension was defined when systolic and/or diastolic blood pressure was > 139 and > 89 mmHg respectively, and/or if the individual was previously diagnosed or on anti-hypertensive medication. Odds Ratios (OR) estimated the within-couple concordance of hypertension while adjusting for five environmental risk factors of hypertension: individual-level body mass index, education and caste, and household-level wealth and place of residence.

**Result:**

OR marginally attenuated from 1.84 (95% Confidence Interval: 1.77, 1.92) to 1.75 (1.68, 1.83) after adjustment, signifying negligible explanation by environmental risk factors, and plausibility of “novel” risk factors. Concordance continued to weaken with age (OR: 2.25 (2.02, 2.52) in < 30 versus 1.36 (1.20, 1.53) in ≥ 60 years).

**Conclusion:**

Our study underscores two critical knowledge gaps: first, the identity of “novel” risk factors of hypertension and second, the mechanism behind weakening of concordance with age. Future research should explore these novel risk factors rigorously and try to modify them. Also, primary healthcare policy of the country should focus on couples in addition to individuals for hypertension and cardiovascular disease screening and management.

## Introduction

Hypertension is one of the major risk factors for cardiovascular disease and premature deaths worldwide. In India, prevalence of hypertension among adults is 29.8% [1] as compared to 31.1% globally [2] and is on the rise. Both genetic and environmental factors predispose individuals to hypertension [3–6], but the contribution of genes is believed to be significantly smaller than the environmental factors [7]. Meanwhile, hypertension has been reported to be clustered between spouses [8], and because spouses are usually genetically unrelated, therefore, spousal concordance is believed to reflect the magnitude of the effect of proximal (high dietary salt intake, physical activities, body mass index, alcohol consumption, smoking etc.) and distal (socio-economic status, education, place of residence etc.) environmental aetiologies of hypertension [9]. These environmental risk factors may aggregate in couples through selection of spouses of same socio-economic class (social homogamy) or [6, 10, 11] with similar attributes and behaviours as one’s own (assortative mating); in a nutshell “likes marrying likes”. The other mechanism of clustering is cohabitation in the shared socio-behavioural environment after marriage.

Until now, spousal resemblances of diseases including hypertension were mainly studied in white Caucasoid samples in western high income nations as evident from studies included in two seminal reviews of spousal aggregation of cardiovascular risk factors and diseases [10, 12]. However, the institution of marriage and relationship between spouses are very different in high-income western nations from low and middle-income countries (LMIC) like India [13]. Unlike in the west, marriage in India is almost universal (everyone gets married), early in life and almost always for life (divorce is very rare); and most importantly, partners in India are mostly selected or “arranged” by parents and not by spouses themselves. And, caste and class homogamy are extensively practiced in such “arranged” marriages [13, 14]. At the same time, unlike west, certain high-risk behaviours, such as tobacco use (smoking and smokeless) and alcohol drinking, are very infrequent in Indian women as opposed to their male counterparts [15]; and also, participation of Indian women in workforce is among the lowest in the world [16]. Therefore, many proximal environmental risk factors of hypertension, such as drinking, smoking and those which are occupation-related are unlikely to cluster in Indian couples in contrast to the west, where the risk factors tend to aggregate in couples as they self-select spouses with similar behaviours. Also, the norms of cohabitation may be different between the two societies, because unlike the west significant differences exist between Indian males and females regarding their roles and agency in family life. In addition to all these differences, the socio-economic pattern of hypertension is also completely different in India, because greater wealth, higher education, social privilege and urban residence are associated with both hypertension and its precursor overweight/obesity in the Indian society whereas it is just the opposite in high-income western nations [17–19].

To our knowledge, spousal concordance of hypertension has not yet been studied in Indians, although recently, few reports of spousal resemblances of diseases have started emerging from Asian populations such as Japan, Korea and China. [20–22]. But, these far eastern nations are relatively affluent and “western”, lifestyle-wise, as compared to India.

Given the stark difference between Indian and the other western and westernised societies, and paucity of evidence from India in this domain, we considered studying spousal concordance of hypertension in India in search for new India-specific environmental risk factors and to inform future hypertension-screening policies (and screening policies for other diseases downstream to hypertension) of the country. Therefore, the current study aimed to comprehensively examine the spousal concordance of hypertension in Indian adults.

## Method

### Data

The study analysed of data of 5^th^ round of National and Family Health Survey (hereinafter NFHS) and 1^st^ wave of Longitudinal Ageing Study in India (hereinafter LASI).

National Family Health Survey (NFHS) is the Indian equivalent of Demographic and Health Survey (DHS), which is periodically conducted in many countries worldwide. The fifth round of NFHS collected information from a nationally representative sample of 636,699 Indian households which were selected using a multistage stratified random cluster sampling design; 724,115 women aged 15–49 years and 101,839 men aged 15–54 years were interviewed between 2019–2021. The response rate was 96.9% among females and 91.6% among males [23].

LASI is the world’s largest and India’s first longitudinal ageing study and is harmonized with other studies that belong to the family of Health and Retirement Surveys, aimed at enabling national estimation and cross-national comparison of indicators related to ageing and health, economic transitions, demographic, and social behaviours in later life [24]. LASI was carried out in 2017 in all 29 Indian states (except Sikkim) and 6 union territories following the sampling strategy of NFHS, which used multistage stratified area probability cluster sampling design. The response rate of LASI was 87.3%.

**Fig. 1:**
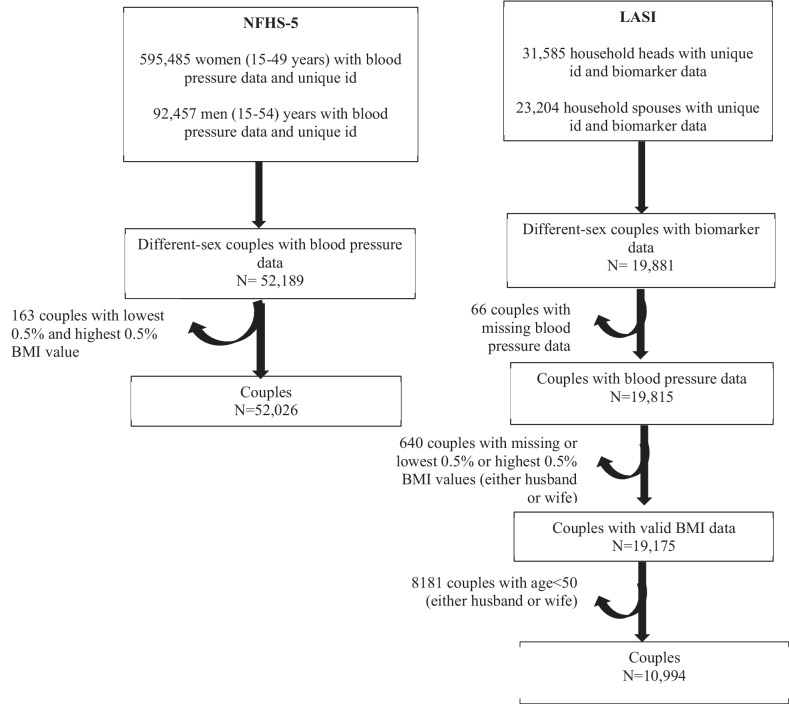
Schematic flow diagram illustrating sample selection

The NFHS comprised primarily a sample of nationally representative Indian women of reproductive age, 15–49 years, and a sub-sample of their spouses of any age. Whereas, LASI represented s sample of Indian adults aged 45 years and above and their spouses of any age. We aimed to study spousal concordance of hypertensions across all age groups and for that we decided to combine these two datasets. To avoid age overlap between samples, we excluded NFHS individuals who were > 49 years and LASI individuals who < 50 years. Consequently, our combined sample comprised 63,020 couples aged 15 years or more and with complete data for all covariates; 52,026 from NFHS aged 15–49 years and 10,994 from LASI aged 50 or more (Fig. 1).

### Measurement

Blood pressure (BP) was measured when respondents were seated and relaxed with legs uncrossed and flat on the floor, left arm with single layer of clothing. Cuff of appropriate circumference was used 1 cm above the elbow with arm held steadily on a flat surface with palm facing up and the centre of the arm at the level of the heart. Three BP readings were recorded with a one min gap in between with the help of the OMRON BP Monitor (Omron HEM 7121); the average of the last two readings was calculated [24]. Though there were three records of BP, the first record of diastolic BP of all females in NFHS had some errors and had to be rejected. Hence, to maintain uniformity, the first BP record was discarded for LASI as well. Hypertension (Y/N) was defined when an average of two measured systolic blood pressure exceeded 139 and /or an average of two measured diastolic blood pressure exceeded89 mm and/or the individual either self-reported to have been previously diagnosed as hypertensive by healthcare personnel or to be receiving anti-hypertensive medications [25].

Gender was a dichotomous variable (male/female) and age was recorded in completed years (wife’s age later categorized, see below). The place of residence was also dichotomous (rural/urban). In NFHS, 26 household assets, domestic fuel use, toilet access, landholding and water sources were composited into a wealth index by principal component analysis and the first component quintiled to divide the sample into 5 groups (poorer, poor, middle class, rich, richer) based on their economic status. Whereas, monthly per capita consumption expenditure (MPCE) quintile was a proxy for household economic status in LASI.

Body Mass Index (BMI) was calculated as measured weight(kg) divided by measured height(m) squared. To remove outlier values of BMI, we removed the top 0.5% (99.5^th^ percentile) and bottom 0.5% (0.5^th^ percentile) observations and then categorized into “obese” (BMI > 30 kg/m2), “overweight” (25–29.9), “normal” (18.5–24.9) and “underweight” (< 18.5). Education was categorized into five categories, “never attended school”, “1–4 years of completed education”, “5–9 years of completed education”, “10–12 years of completed education”, “ > 12 years of completed education”. Caste was categorized into “Scheduled Caste”, “Scheduled Tribe”, “Other Backward Class”, “General”, and “Others”.

### Statistical analysis

#### Descriptive statistics

Spouse-level (individual-level) variables age, caste, BMI status, education and hypertension were summarized by their frequency (percentage) in husbands and wives separately. Resemblance between spouses were initially explored by examining whether both belonged to the same categories of these variables or not. For couple-level (household-level) variables, wealth and place of residence, frequency (percentage) was expressed at the level of couples.

#### Estimation of spousal concordance and covariate adjustment

In spousal studies, researchers have mostly strived to estimate the contribution of the conventional environmental risk factors to concordance of hypertension. For this, most have statistically adjusted for classical individual-level risk factors between spouses, such as BMI and education and household-level risk factors, such as wealth and rural/urban residence. Further, few authors had stratified their analyses by length of marriage (or a surrogate of length of marriage which is often the wife’s or husband’s age), positing that increasing spousal concordance with age would imply cohabitation being the more dominant mechanism than selection of similar spouse in aggregation of hypertension (and its risk factors) within couples [10, 12, 26].

We first computed Odds Ratios (OR) from logistic regression models to estimate the association between hypertension status of husbands (principal independent variable) and wives (dependent variable). We then adjusted our models for both wives’ and husbands’ caste, education and BMI, and household-level wealth and place of residence – the five conventional environmental risk factors of hypertension in the Indian context. We did not adjust for tobacco and alcohol use as they were present in very few Indian wives, unlike the situation in high-income nations. We used wives’ age as a surrogate for length of marriage and created five categories of couples whose wives’ ages were < 30, 30–39, 40–49, 50–59 and > 59 years (length of marriage was neither present in LASI nor in NFHS, hence the use of surrogate) as other researchers of this domain have done [27–29]. Adjusted ORs were then estimated across these five age-groups to examine changes in the strength of concordance with age.

In addition to statistical co-variate adjustment, “restricting” analyses to certain strata of co-variates is another robust method of controlling for their effects [30]. Therefore, our analysis was restricted to the low-risk strata of the five co-variates: first among less wealthy, second among rural couples, third among couples where both the spouses were less educated, fourth where both the spouses were of underprivileged caste and fifth where both the spouses were of normal/under-weight category. Lastly, the analysis was conducted in a sub-population belonging to the low-risk strata for all the five co-variates simultaneously. To get a large enough sample for the last set of models, the oldest two age-groups (50–59 and ≥ 60) were amalgamated.

All analyses were performed using Stata statistical software version 15.0 [31]. The descriptive statistics used sampling weights to account for unequal probability of selection, stratification and non-response. But we did not apply sample weights in the logistic regression models, same as what others have also done with regards to explanatory modelling [32].

## Result

The sample, approximating the Indian adult population, was predominantly rural (75%). Slightly more than half of the respondents were < 40 years old and a quarter had not attended school (almost 70% had up to upper primary schooling only). More than 60% of the respondents had normal BMI, with only 12% and 5% being underweight and obese respectively. Members of Other Backward Caste comprised 41% of the sample, the Scheduled Caste (19%), Scheduled Tribe (21%) and General Caste (20%) equally making up the rest. Prevalence of hypertension was 28% (Table 1).

**Table 1:** Sample characteristics

**Variables**	**Individual spousal characteristics**			**Couple characteristics**	
	**Husband (n, %)**	**Wife (n, %)**	**Total**	**Concordant/Discordant couples (n, %)**	
	***n* = 63,020**	***n* = 63,020**	**(*n* = 126,040)**	***n* = 63,020**	
**Hypertension**					
Hypertensive	19,949 (31.66%)	14,873 (23.60%)	34,822 (28%)	Both hypertensive	7152 (11.35%)
Non hypertensive	43,071 (68.34%)	48,147 (76.40%)	91,218 (72%)	Both not hypertensive	35,350 (56.06%)
				Hypertension status- discordant couples	20,518 (32.56%)
**Age**					
< 30	9342 (14.82%)	18,003 (28.57%)	27,345 (22%)	Both < 30	9083 (14.41%)
30–39	19,207 (30.48%)	20,020 (31.77%)	39,227 (31%)	Both 30–39	10,630 (16.87%)
40–49	17,479 (27.74%)	14,003 (22.22%)	31,482 (25%)	Both 40–49	8250 (13.09%)
50–59	8628 (13.69%)	5900 (9.36%)	14,528 (12%)	Both 50–59	2581 (4.10%)
> 60	8364 (13.27%)	5094 (8.08%)	13,458 (11%)	Both > 60	5045 (8.01%)
				Age- discordant couples	27,451 (43.53%)
**Completed years of Education (years)**					
Never attended school	12,094 (19.19%)	21,321 (33.83%)	33,415 (27%)	Both never attended school	9296 (14.75%)
Till primary education (Up to 5 years)	10,203 (16.19%)	9300 (14.76%)	19,503 (15%)	Both up to primary education (Up to 5 years)	2683 (4.26%)
Upper primary (Between 6–9 years)	17,126 (27.18%)	14,696 (23.32%)	31,822 (25%)	Both upper primary (Between 6–9 years)	6037 (9.58%)
Secondary and higher secondary (between 10–12 years)	15,320 (24.31%)	11,961 (18.98%)	27,281 (22%)	Both secondary and higher secondary (between 10–12 years)	5547 (8.80%)
Graduate and above (> 12 years)	8277 (13.13%)	5742 (9.11%)	14,019 (11%)	Both graduate and above (> 12 years)	3608 (5.73%)
				Education- discordant couples	35,849 (56.89%)
**Body Mass Index (BMI)**					
< 18.5 kg/m2	6522 (10.44%)	8630 (13.80%)	15,152 (12%)	Both < 18.5 kg/m2	1850 (2.98%)
18.5–24.99 kg/m2	39,038 (62.46%)	36,805 (58.87%)	75,843 (61%)	Both 18.5–24.99 kg/m2	24,356 (39.27%)
25.0–29.99 kg/m2	14,466 (23.15%)	12,912 (20.65%)	27,378 (22%)	Both 25.0–29.99 kg/m2	4252 (6.86%)
≥ 30 kg/m2	2470 (3.95%)	4175 (6.68%)	6645 (5%)	Both ≥ 30 kg/m2	468 (0.75%)
				BMI- discordant couples	32,094 (50.93%)
**Caste**					
Scheduled Tribe	12,363 (19.62%)	12,400 (19.68%)	24,763 (21%)	Both Scheduled Tribe	9542 (15.14%)
Scheduled Caste	11,570 (18.36%)	11,517 (18.28%)	23,087 (19%)	Both Scheduled Caste	11,240 (17.84%)
Other Backward Caste	24,423 (38.75%)	24,391 (38.70%)	48,814 (41%)	Both Other Backward Caste	21,378 (33.92%)
General Caste	11,951 (18.96%)	11,684 (18.54%)	23,635 (20%)	Both General Caste	9560 (15.17%)
No caste or didn't know	2713 (4.30%)	3028 (4.80%)	5741 (5%)	Both no caste or didn't know	1784 (2.83%)
				Caste-discordant couples	9516, 15.10%
**Wealth quintiles**					
Poorest	NA				12,932 (20.52%)
Poorer					13,725 (21.78%)
Middle					13,235 (21.00%)
Richer					12,351 (19.60%)
Richest					10,777 (17.10%)
**Residence**					
Rural	NA				46,965 (74.52%)
Urban					16,055 (25.48%)

Wives were significantly younger (29% wives < 30 years versus 15% husbands) and had less educational attainment than their husbands (34% wives never attending school versus 19% husbands). Wives were also slightly more likely to be underweight as well as obese in comparison to their husbands. Hypertension was more prevalent in the males (32% in husbands versus 24% in wives) (Table 1).

Fifty six percent couples had husbands and wives belonging to the same age group, whereas educational concordance was observed in 43% couples. Almost half of the couples had similar BMI whereas caste homogamy was frequent (85% couples having spouses from the same caste). Both the partners were hypertensive in 11% couples, with 33% couples having only one spouse suffering from hypertension. (Table 1).

In the overall sample, the odds of hypertension were 1.84 times more in wives with hypertensive husbands as compared to wives with normotensive husbands. The odds ratio (OR) only modestly attenuated to 1.75 after controlling for the environmental risk factors of hypertension that included spouse-level age, education, caste and BMI and couple-level wealth and place of residence (Table 2).

**Table 2:** Estimation of spousal concordance of hypertension in different strata across different age groups

**Variable**	**Overall** ***n* = 63,020**		**In couples with low wealth** **(household wealth in lowest two quintiles)** ***n* = 26,657**	**In rural couples** **(Household located in rural area)** ***n* = 46,965**	**In couples with low educational attainment** **(both either illiterate or maximum primary education)** ***n* = 35,378**	**In couples with normal/low BMI** **(both < 25 kg/m**^**2**^**)** ***n* = 35,790**	**In couples of underprivileged castes** **(both underprivileged caste)** ***n* = 21,921**	**In rural couples with low wealth, low educational attainment, low BMI and of underprivileged caste** ***n* = 4243**
	**OR (95% CI)**	**adjusted**^a^ **OR (95% CI)**	**adjusted**^b^ **OR (95% CI)**	**adjusted**^b^ **OR (95% CI)**	**adjusted**^b^ **OR (95% CI)**	**adjusted**^b^ **OR (95% CI)**	**adjusted**^b^ **OR (95% CI)**	**OR (95% CI)**
**All age-groups combined**								
	1.84 (1.77, 1.92)	1.75 (1.68, 1.83)	1.83 (1.71, 1.96)	1.82 (1.73, 1.92)	1.72 (1.59, 1.85)	1.88 (1.77, 1.99)	1.81 (1.68, 1.94)	1.97 (1.68, 2.31)
**Stratified by wife’s age**								
**< 30**	2.27 (2.05, 2.55)	2.25 (2.02, 2.52)	2.55 (2.15, 3.02)	2.38 (2.10, 2.70)	2.25 (1.67, 3.02)	2.49 (2.16, 2.88)	2.14 (1.78, 2.58)	3.3 (1.99, 5.47)
**30–39**	2.10 (1.95, 2.26)	2.03 (1.89, 2.20)	2.12 (1.88, 2.40)	2.08 (1.91, 2.29)	2.25 (1.91, 2.64)	2.30 (2.06, 2.58)	2.23 (1.96, 2.55)	2.36 (1.74, 3.21)
**40–49**	1.74 (1.62, 1.87)	1.66 (1.53, 1.79)	1.65 (1.46, 1.88)	1.72 (1.58, 1.89)	1.75 (1.53, 1.99)	1.73 (1.54, 1.95)	1.67 (1.47, 1.91)	1.55 (1.17, 2.06)
**50–59**	1.65 (1.49, 1.84)	1.52 (1.36, 1.69)	1.65 (1.39, 1.97)	1.56 (1.36, 1.78)	1.56 (1.33, 1.83)	1.55 (1.34, 1.80)	1.53 (1.26, 1.85)	1.95 (1.47, 2.58)
**> 60**	1.58 (1.42, 1.77)	1.36 (1.20, 1.53)	1.47 (1.22, 1.78)	1.42 (1.23, 1.64)	1.43 (1.22, 1.68)	1.49 (1.29, 1.73)	1.42 (1.146, 1.76)	

^a^Adjusted for wealth, rural/urban residence, educational attainment, BMI, caste

^b^Adjusted for other four variables except the one used for stratification

**Fig. 2:**
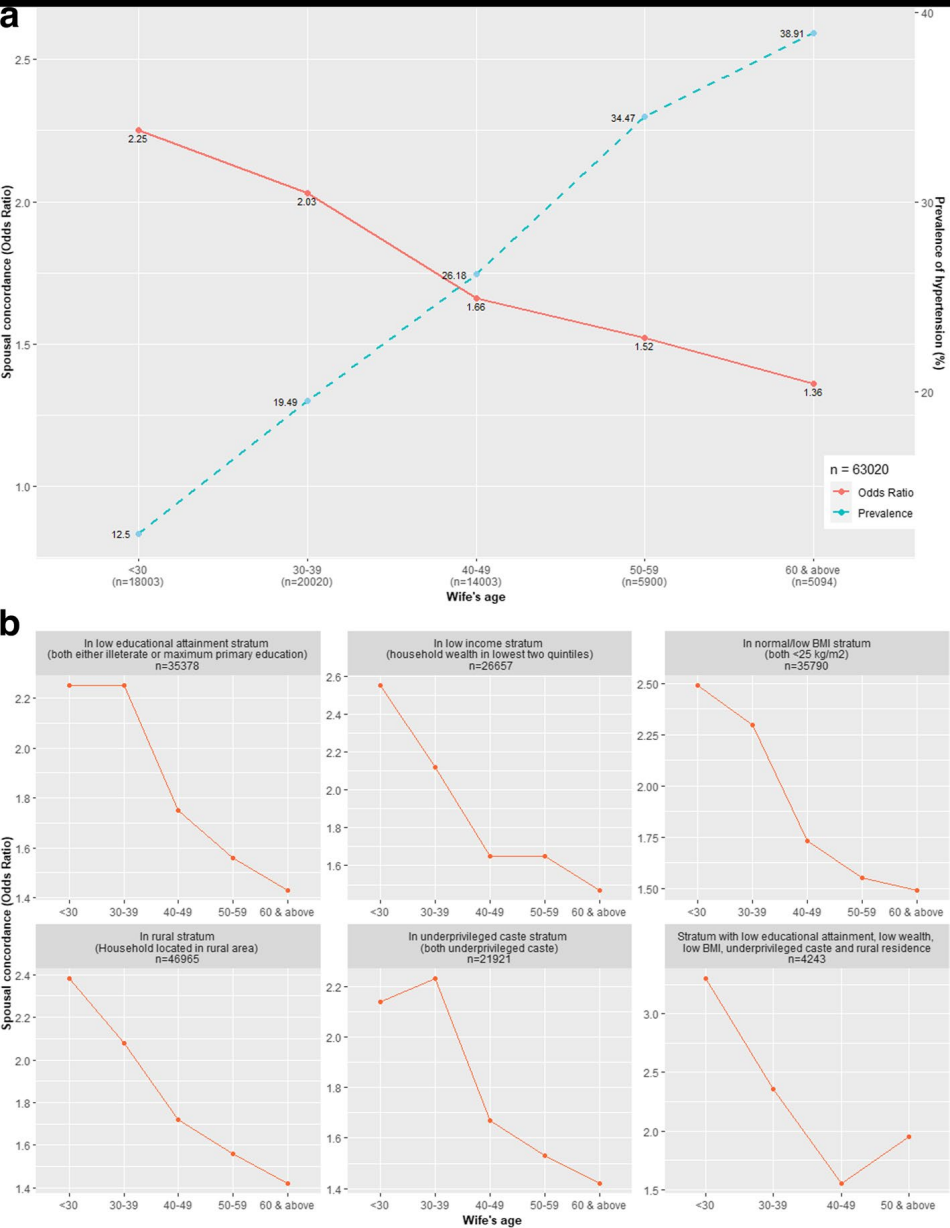
**a** and **b** Age-wise changes in spousal concordance of hypertension in entire sample and across different strata

The prevalence of hypertension kept rising (12.5% in < 30 years to 38.9% in ≥ 60 years) with wives’ age. However, spousal concordance of hypertension (OR from 2.25 in < 30 years group to 1.36 in ≥ 60 years age group) continued to weaken significantly as prevalence of hypertension surged with age (Fig. 2a). The rise of hypertension and weakening of concordance maintained a monotonous pattern across the age-groups (Fig. 2b). The estimates and the monotony of patterns hardly changed after adjustment with the conventional environmental risk factors. (Table 2).

In the low-risk strata of education (illiterate or less than primary education), wealth (lowest two quintile of wealth), BMI (low or normal), place of residence (rural) and caste (SC and ST), concordance patterns, almost similar to the overall sample, could be observed. For instance, in low wealth, low education, low/normal BMI, less privileged caste and rural residence strata, the ORs of spousal concordance respectively were 2.55, 2.25, 2.49, 2.14 and 2.38 while the wife’s age was < 30 as compared to 1.47, 1.43, 1.49, 1.42 and 1.42 when the wife was ≥ 60 years old. Even in a sub-sample considering only the low-risk strata of all the five risk factors simultaneously, a similar pattern of spousal concordance was evident (OR at < 30 years and ≥ 50 years were 3.30 and 1.95 respectively) (Fig. 2b).

## Discussion

In a large nationally representative sample of Indian adults, wives living with hypertensive husbands were significantly more likely to suffer from hypertension themselves as compared to wives living with normotensive husbands. Notably, the younger couples displayed stronger concordance of hypertension when hypertension is relatively less common; and the concordance grew weaker in older age groups as the prevalence of hypertension started increasing with age.

As per the existing knowledge in this field, first, clustering of hypertension in couples is due to clustering of conventional environmental risk factors of hypertension in them [10, 12]. Second, these environmental risk factors either cluster through selection of similar spouse (through social homogamy or assortative mating) or post-marital cohabitation in the same environment; and weakening of concordance with duration of marriage implies that these risk factors had mainly clustered through spouse selection and not through post-marital cohabitation [26]. However, the evidence from our data contradicts both of these. First, adjustment with conventional environmental risk factors of hypertension – greater wealth, higher education, privileged caste, urban residence, and excess bodyweight – did not explain much of the concordance in our sample, attenuation of OR from 1.84 to 1.75 signifying only 12% explanation of concordance by these co-variates. Also, most notably, strong concordance was also observed in the strata with lowest socio-economic risk for hypertension. Therefore, one may conclude that the clustering of conventional environmental and socio-economic risk factors were not the main drivers of concordance of hypertension between Indian spouses. Also, as because the socio-economic variables were not the main drivers of concordance, therefore, selection of similar spouse (social homogamy or assortative mating) was perhaps not the dominant mechanism of concordance in our sample, although we found concordance to weaken with wife’s age.

So, two critical knowledge gaps emerge from our analyses which are framed below as “unanswered questions”:

- If not the conventional environmental risk factors, then what may be the other risk factors that cluster in Indian couples causing spousal concordance of hypertension in them?
- What is the plausible explanation for stronger concordance in young Indian couples (when hypertension is less common) and its weakening with age (given the concordance is unlikely to be entirely driven by social homogamy or assortative mating)?

We posit that “novel” risk factors of hypertension, present in the air, water (for example pollutants) [33–35], diet or the psycho-social environment of the family, unlinked to socio-economic status, might be aggregating in Indian couples in their early married lives through cohabitation. The likely explanation for stronger concordance in early adult life is that there is greater cohabitation-led sharing of household environment between young spouses. But increasing divergence of roles with age between husband and wives in the Indian society leads to less sharing of household environment and hence, divergence of their risk profile and therefore, weakening of concordance in later life. These hypothesized novel risk factors may also underlie the resemblance in concordance patterns observed in two completely different societies, India and The Netherlands [26].

In the absence of any alternate explanation, the hypothesis of novel environmental risk factors aggregating through cohabitations seems highly plausible. In this connection it would be worth mentioning that Speers et al. wrote in 1989 “Based on these findings, future research on spousal concordance on blood pressure needs to explore new variables, those that might illuminate the unidentified shared environment that accounts for the spousal concordance in blood pressure” [36]. The field has not progressed much since then regarding identification of new risk factors of hypertension, although there is a possibility that these unidentified risk factors may pose a considerable burden of hypertension and its cardiovascular sequelae on the Indian society. Therefore, future research should try to bridge this significant knowledge gap by unravelling the novel aetiologies of hypertension, many of which may be modifiable. The spousal study design with our customization of stratified analysis can be a perfect template for this; whereby various novel exposures of concordant couples may be compared with discordant couples in different socio-economic strata.

The extant literature in this domain is rife with ambiguity also, for example, few studies have reported complete explanation of spousal concordance by conventional risk factors, whereas others identified substantial concordance independent of these determinants [8, 37]. Similarly, the evidence on the role of length of marriage (or spousal age) in moderating the spousal resemblance is ambiguous, as noted by a review [12, 20]. Few reported increases (which had given rise to the idea that cohabitation may have a stronger effect than assortative mating on spousal concordance), few reported decreases and few reported no changes in concordance estimates with rising length of marriage.

Most of these spousal studies from the past were limited by their smaller sample sizes [26], thus perhaps not having adequate statistical power to identify spousal concordance after co-variate adjustment or stratification. In contrast, the strength of our study lies in its size and span, covering the entire adult age spectrum, because we could use two national samples, one representing 15–49 years and the other representing 50 + years Indians. Also, the blood pressure was objectively measured in both the studies that could identify the undiagnosed hypertensives, a distinct improvement on many previous spousal studies that included only self-reported patients of hypertension [10, 12]– often a source of serious misclassification bias. The other strength of our study is the methodology, as we conducted both statistical adjustment and stratified analysis to account for the effect of environmental risk factors and both the approaches led to similar conclusions, thereby strengthening our study findings considerably.

Our study has few limitations, perhaps all the environmental risk factors of hypertension could not be accounted for in the analyses. For instance, we could not adjust for dietary salt or physical activity, as these were not measured in both the surveys. Arguably, these could have explained some of the concordance of hypertension within couples. But, it is unlikely that the effect of these variables would be so large that it would explain the entire “unexplained” concordance as Speers et al. had also mentioned before [36, 38]. Moreover, body mass index, the proxy variable for physical activity was adjusted for in the analysis, but it failed to explain much of the concordance. Also, we did not adjust for smoking and alcohol consumption, because as mentioned earlier, the rates for the use of these substances are very less among Indian women as compared to men, so their chance of clustering within couples leading to concordance of hypertension were minimal. We also did not adjust for occupation, because Indian women in paid occupation is much less than men and substantial clustering of occupation between spouses is unlikely in the Indian context to explain concordance of hypertension. We also considered wife’s age as surrogate for length of marriage as others have also done in this domain [27–29]. Moreover, the institution of marriage being universal in India and relatively early in life, wife’s age in the Indian context is a reasonable proxy for marriage duration. However, future large population surveys of India may consider capturing the length of marriage, as it can embody some valuable information for different studies.

On the policy front, India is currently gearing up for large scale screening and treatment of chronic conditions like hypertension and diabetes through its primary healthcare system, which is currently undergoing a major overhaul under the signature health system strengthening scheme of the country called *Ayushman Bharat* [39]. Also, India launched Indian Hypertension Control Initiative (IHCI) [40] in 2017 to achieve 25% relative reduction hypertension by 2025. As we had observed substantial spousal concordance of hypertension – a very common chronic condition of late adult life and a critical precursor of cardiovascular diseases– it is recommended that the future strategy should consider screening the spouse whenever a hypertensive is identified. Our study results also call for primordial or primary prevention strategies to be targeted at couples rather than two separate individuals, because existing evidence shows spouses reinforce favourable lifestyle changes in each other [22, 25].

To conclude, our study has shown perhaps for the first time from India that there is significant spousal concordance of hypertension unexplained by clustering of conventional environmental risk factors such as greater wealth, higher education, privileged caste, urban residence and excess bodyweight in couples. The spousal concordance is stronger in younger couples and consistently weakens with age. We realize that there is a critical knowledge gap regarding the actual drivers of this concordance and hence we hypothesize that this observed concordance may be due to novel environmental risk factors present in the shared household environment where the spouses cohabit. Future research should explore these novel risk factors rigorously and try to modify them. Also, primary healthcare policy of the country should focus on couples instead of individuals for hypertension and cardiovascular disease screening and management.

## Data Availability

Publicly available datasets were used and/or analysed for the current study. LASI—Data | International Institute for Population Sciences (IIPS) (iipsindia.ac.in). The DHS Program—India: Standard DHS, 2019–21 Dataset.

## Abbreviations

LASI: Longitudinal Ageing Study in India Wave
NFHS: National Family Health Survey
OR: Odds Ratios
LMIC: Low and middle-income countries
MPCE: Monthly per capita consumption expenditure
BP: Blood Pressure
BMI: Body Mass Index

## References

[1] Anchala R, Kannuri NK, Pant H, Khan H, Franco OH, Di Angelantonio E (2014). Hypertension in India: a systematic review and meta-analysis of prevalence, awareness, and control of hypertension. J Hypertension.

[2] Mills KT, Stefanescu A, He J. The global epidemiology of hypertension. Nat Rev Nephrol Nature Research. 2020;16:223–37. Available from: https://www.nature.com/articles/s41581-019-0244-2. [Cited 2021 Apr 17]. 10.1038/s41581-019-0244-2 PMC7998524 32024986

[3] Warren HR, Evangelou E, Cabrera CP, Gao H, Ren M, Mifsud B, et al. Genome-wide association analysis identifies novel blood pressure loci and offers biological insights into cardiovascular risk. Nat Genet. 2017;49(3):403–15. Available from: https://www.nature.com/articles/ng.3768. [Cited 2021 Apr 17]. 10.1038/ng.3768 PMC5972004 28135244

[4] Pazoki R, Dehghan A, Evangelou E, Warren H, Gao H, Caulfield M, et al. Genetic predisposition to high blood pressure and lifestyle factors: associations with midlife blood pressure levels and cardiovascular events. Circulation. 2018;137(7):653–61. Available from: https://www.ahajournals.org/doi/abs/10.1161/CIRCULATIONAHA.117.030898. [Cited 2021 Apr 17]. 10.1161/CIRCULATIONAHA.117.030898 29254930

[5] Hypertension: MedlinePlus Genetics. Available from: https://medlineplus.gov/genetics/condition/hypertension/#causes. [Cited 2021 Apr 17].

[6] Sackett DL, Anderson GD, Milner R, Feinleib M, Kannel WB (1975). Concordance for coronary risk factors among spouses. Circulation.

[7] Biino G, Parati G, Concas MP, Adamo M, Angius A, Vaccargiu S, et al. Environmental and Genetic Contribution to Hypertension Prevalence: Data from an Epidemiological Survey on Sardinian Genetic Isolates. PLoS One 2013;8(3). Available from: https://www.ncbi.nlm.nih.gov/pmc/articles/PMC3603911/. [Cited 2021 Apr 19]. 10.1371/journal.pone.0059612 PMC3603911 23527229

[8] Hippisley-Cox J, Pringle M (1998). Are spouses of patients with hypertension at increased risk of having hypertension? A population-based case-control study. Br J Gen Pract.

[9] Dufouil C, Alpérovitch A (2000). Couple similarities for cognitive functions and psychological health. J Clin Epidemiol.

[10] Di Castelnuovo A, Quacquaruccio G, Donati MB, De Gaetano G, Iacoviello L (2009). Spousal concordance for major coronary risk factors: a systematic review and meta-analysis. Am J Epidemiol.

[11] Knuiman MW, Divitini ML, Bartholomew HC, Welborn TA (1996). Spouse correlations in cardiovascular risk factors and the effect of marriage duration. Am J Epidemiol.

[12] Wang Z, Ji W, Song Y, Li J, Shen Y, Zheng H, et al. Spousal concordance for hypertension: a meta-analysis of observational studies. J Clin Hypertens. 2017;19(11):1088–95. Available from: http://doi.wiley.com/10.1111/jch.13084. [Cited 2021 Mar 23]. 10.1111/jch.13084 PMC8030758 28856830

[13] Sharma I, Pandit B, Pathak A, Sharma R. Hinduism, marriage and mental illness. Indian J Psychiatry. 2013;55:S243. Wolters Kluwer -- Medknow Publications. Available from: https://www.ncbi.nlm.nih.gov/pmc/articles/PMC3705690/. [Cited 2021 Apr 19]. 10.4103/0019-5545.105544 PMC3705690 23858262

[14] Prakash R, Singh A. Who marries whom? Changing mate selection preferences in Urban India and emerging implications on social institutions. Popul Res Policy Rev. 2014;33(2):205–27. Available from: http://link.springer.com/10.1007/s11113-013-9294-5.

[15] Goel S, Tripathy JP, Singh RJ, Lal P (2014). Smoking trends among women in India: Analysis of nationally representative surveys (1993–2009). South Asian J Cancer..

[16] MOSPI. eriodic Labour Force Survey (PLFS), 2019–2020. 2022. p. 1. Available from: https://pib.gov.in/PressReleasePage.aspx?PRID=1805783. [Cited 2022 Nov 28].

[17] Corsi DJ, Subramanian SV. Socioeconomic gradients and distribution of diabetes, hypertension, and obesity in India. JAMA Netw Open. 2019;2(4):e190411. Available from: https://www.ncbi.nlm.nih.gov/pmc/articles/PMC6450330/?report=abstract. [Cited 2020 Aug 18]. 10.1001/jamanetworkopen.2019.0411 PMC6450330 30951154

[18] Bhansali A, Dhandania VK, Deepa M, Anjana RM, Joshi SR, Joshi PP (2015). Prevalence of and risk factors for hypertension in urban and rural India: the ICMR-INDIAB study. J Hum Hypertens.

[19] Dutta A, Kavitha AK, Samal S, Panigrahi P, Swain S, Nanda L (2018). Independent urban effect on hypertension of older Indians: identification of a knowledge gap from a Study on Global AGEing and Health. J Am Soc Hypertens.

[20] Jurj AL, Wen W, Li HL, Zheng W, Yang G, Xiang YB (2006). Spousal correlations for lifestyle factors and selected diseases in Chinese couples. Ann Epidemiol.

[21] Chen X, Hu X, Shi S, Tian Q. Socioeconomic and demographic factors for spousal resemblance in obesity status in China. Healthcare. 2020;8(4):415. Available from: https://www.ncbi.nlm.nih.gov/pmc/articles/PMC7711872/. [Cited 2021 Apr 17]. 10.3390/healthcare8040415 PMC7711872 33096592

[22] Kang S, Kim M, Won CW. Spousal concordance of physical frailty in older Korean couples. Int J Environ Res Public Health. 2020;17(12):1–10. Available from: https://www.ncbi.nlm.nih.gov/pmc/articles/PMC7344744/. [Cited 2021 Apr 19]. 10.3390/ijerph17124574 PMC7344744 32630401

[23] IIPS/India II for PS, ICF. India national family health survey NFHS-5 2019-21. 2022. Available from: https://dhsprogram.com/publications/publication-FR375-DHS-Final-Reports.cfm. Accessed 26 2023 Jul.

[24] International Institute for Population Sciences (IIPS). Longitudinal Ageing Study in India (LASI). Fact Sheet. 2010; Available from: http://iipsindia.org/research_lasi.htm.

[25] Demarco MMA, Coresh J, Woodward M, Butler KR, Kao WHL, Mosley TH, et al. Hypertension status, treatment, and control among spousal pairs in a middle-aged adult cohort. Am J Epidemiol. 2011;174(7):790–6. Available from: https://www.ncbi.nlm.nih.gov/pmc/articles/PMC3203378/. [Cited 2021 Apr 9]. 10.1093/aje/kwr167 PMC3203378 21841158

[26] Nakaya N, Xie T, Scheerder B, Tsuchiya N, Narita A, Nakamura T, et al. Spousal similarities in cardiometabolic risk factors: a cross-sectional comparison between Dutch and Japanese data from two large biobank studies. Atherosclerosis. 2021;334:85–92. Available from: https://linkinghub.elsevier.com/retrieve/pii/S0021915021013101. 10.1016/j.atherosclerosis.2021.08.037 34492521

[27] Longini IM, Higgins MW, Hinton PC, Moll PP, Keller JB (1984). Environmental and genetic sources of familial aggregation of blood pressure in Tecumseh. Michigan. Am J Epidemiol..

[28] Hayes CG (1971). Family Aggregation of Blood Pressure in Evans County, Georgia. Arch Intern Med..

[29] Suarez L, Criqui MH, Barrett-Connor E (1983). Spouse concordance for systolic and diastolic blood pressure. Am J Epidemiol.

[30] Jager KJ, Zoccali C, MacLeod A, Dekker FW (2008). Confounding: what it is and how to deal with it. Kidney Int..

[31] Corp S (2017). Stata statistical software: release 15.

[32] Lee J, Wilkens J, Meijer E, Sekher TV, Bloom DE, Hu P (2022). Hypertension awareness, treatment, and control and their association with healthcare access in the middle-aged and older Indian population: a nationwide cohort study. Basu S, editor. PLOS Med..

[33] Giorgini P, Di Giosia P, Grassi D, Rubenfire M, Brook RD, Ferri C. Air pollution exposure and blood pressure: an updated review of the literature. Curr Pharm Des. 2016;22(1):28–51. Available from: http://www.ncbi.nlm.nih.gov/pubmed/26548310. 10.2174/1381612822666151109111712 26548310

[34] Curto A, Wellenius GA, Milà C, Sanchez M, Ranzani O, Marshall JD, et al. Ambient Particulate Air Pollution and Blood Pressure in Peri-urban India. Epidemiology. 2019;30(4):492–500. Available from: http://www.ncbi.nlm.nih.gov/pubmed/31162282. 10.1097/EDE.0000000000001014 PMC6558270 31162282

[35] Choi YJ, Kim SH, Kang SH, Kim SY, Kim OJ, Yoon C-H (2019). Short-term effects of air pollution on blood pressure. Sci Rep..

[36] Speers MA, Kasl SV, Ostfeld AM (1989). Marital correlates of blood pressure. Am J Epidemiol.

[37] Hippisley-Cox J, Coupland C, Pringle M, Crown N, Hammersley V. Married couples’ risk of same disease: cross sectional study. BMJ. 2002;325(7365):636. Available from: https://www.bmj.com/lookup/doi/10.1136/bmj.325.7365.636. [Cited 2021 Mar 23]. 10.1136/bmj.325.7365.636 PMC126307 12242177

[38] Speers MA, Kasl SV, Freeman DH, Ostfeld AM (1986). Blood pressure concordance between spouses. Am J Epidemiol.

[39] Gopichandran V (2019). Ayushman Bharat National Health Protection Scheme: an Ethical Analysis. Asian Bioeth Rev.

[40] Kaur P, Kunwar A, Sharma M, Durgad K, Gupta S, Bangar SD, et al. The India Hypertension Control Initiative–early outcomes in 26 districts across five states of India, 2018–2020. J Hum Hypertens. 2022. Available from: https://www.nature.com/articles/s41371-022-00742-5. 10.1038/s41371-022-00742-5 PMC10328822 35945426

